# The Incidence of Cholelithiasis After Bariatric Surgery in Saudi Arabia and Its Associated Risk Factors

**DOI:** 10.7759/cureus.40549

**Published:** 2023-06-17

**Authors:** Rawan A Altalhi, Raghad M Alsaqqa, Raghad M Alasmari, Amal Aljuaid, Lama Althobaiti, Mohammad Eid M Mahfouz

**Affiliations:** 1 College of Medicine, Taif University, Taif, SAU

**Keywords:** saudi arabia, incidence, weight loss, gallstones, cholelithiasis, bariatric surgery

## Abstract

Introduction: Saudi Arabia has one of the highest obesity rates (35.4%) in the world, and bariatric surgery (BS) has emerged as the most effective treatment for obesity and its comorbidities. Despite its effectiveness, it is a known risk factor for cholelithiasis. The aim of this study is to identify the incidence and risk factors that contribute to the development of symptomatic cholelithiasis after different types of bariatric surgery in Saudi Arabia.

Methods: This is a cross-sectional study conducted among the Saudi adult population. The sample size was 706 participants who underwent bariatric surgery from all over Saudi Arabia. Data collection was done through a validated online self-reported survey.

Results: Out of 706 participants who fulfilled the inclusion criteria, it was found that the incidence of gallstones (GS) after bariatric surgery was 18.8%. The most incidence was during the first year of surgery, where the number of individuals reached 80.4%. The majority were in females (22.9%) and those who underwent Roux-en-Y gastric bypass (RYGB) surgery (51.2%). Patients who had a body mass index (BMI) of >25 kg/m² significantly had a higher incidence of gallstones (23.1%) compared to those who had a lesser BMI (15.8%). As the analysis showed, the medication used to prevent the occurrence of gallstones can be considered one of the protective factors, where 85.4% of individuals who used these medications did not develop cholelithiasis.

Conclusion: The incidence of gallstones after bariatric surgery was high, particularly within the first year of surgery. The increase in postoperative gallstone formation is correlated with hyperlipidemia and Roux-en-Y gastric bypass as basic predictive factors. On the contrary, the medication used to prevent the occurrence of gallstones is considered a protective factor.

## Introduction

Global obesity prevalence has risen dramatically during the last five decades, reaching pandemic proportions [1]. The most recent data from the World Health Organization (WHO) stated that Saudi Arabia is the 14th most obese country (35.4%) [2]. The major impact of obesity on the health and healthcare system emphasizes the significance of developing appropriate obesity solutions [3].

Regardless of the surgery, the management of obesity is generally ineffectual in long-term weight control [4]. Bariatric surgery (BS) is one of the most effective treatments for morbid obesity, as it significantly reduces and treats obesity-related comorbidities and decreases the risk of death [5,6].

The mechanism of bariatric surgeries depends in different ways, whether by reducing the capacity of the stomach and increasing early satiation, limiting the capability of the body to absorb nutrients, or having a combination of both [7]. Globally, 833,687 operations were performed in 2019 according to the International Federation for the Surgery of Obesity and Metabolic Disorders (IFSO). The most used procedures were sleeve gastrectomy (SG) (47%), Roux-en-Y gastric bypass (RYGB) operations (35.3%), gastric banding procedures (8.4%), and one anastomosis gastric bypass (OAGB) procedures (3.7%) [8].

Despite the effectiveness of bariatric surgery, it has a significant risk factor for cholelithiasis, which can range from asymptomatic to symptomatic cholelithiasis requiring cholecystectomy [9]. Most gallstones (GS) formed in the first two years following surgery [10]. Rapid weight loss in bariatric surgery causes increased cholesterol saturation in the bile, decreases bile acid secretion, increases mucin secretion 10-20-folds, and finally reduces gallbladder emptying that causes bile stasis, all of which are essential contributors to the formation of gallstones [9,11].

Many studies around the world reported the incidence of the development of gallstones after bariatric surgery, which ranges from 30% to 53% [12]. There was a significant difference in the types of bariatric surgery, with the incidence of cholelithiasis in RYGB varying from 49% to 71%, but in SG, the incidence ranges from 29% to 48% [13]. Furthermore, based on two population-based studies, bariatric surgery is associated with a fivefold greater risk of cholelithiasis than the general population [14,15].

In addition, higher and rapid excess weight loss (%EWL) after bariatric surgery has a higher incidence of symptomatic cholelithiasis [16-19]. On the other hand, Manatsathi et al. [10] found no association between gallstone formation and the amount or rate of weight loss.

A recent prospective study showed a high incidence (22.7%) of gallstones after bariatric surgery, and the risk factors were a high percentage of excessive weight loss, rapid weight loss, longer duration of obesity, and gastroesophageal reflux disease (GERD) [20].

In Saudi Arabia, the cholelithiasis incidence after bariatric surgery ranges between 2.3% and 6.53% [12,19,21]. Aldriweesh et al. [19] reported a significant relationship between the amount of weight loss and the formation of gallstones after bariatric surgery, while another study found that the only risk factor was rapid weight loss [12]. Also, both studies denied any association between symptomatic cholelithiasis and age, gender, and other comorbidities such as diabetes mellitus (DM), hypertension (HTN), and dyslipidemia (DLP) [12,19].

The data about gallstone formation after bariatric surgery in Saudi Arabia are limited, and further studies are needed. Therefore, this study aimed to determine the incidence of cholelithiasis after different types of bariatric surgery among the Saudi population and identify the risk factors for cholelithiasis after bariatric surgery.

## Materials and methods

Study design and participants

A cross-sectional study targeted the entire accessible population who have undergone bariatric surgery in Saudi Arabia. Ethical approval has been acquired from the Ethics Committee of Taif University (number 43-078) from November 2021 to November 2022.

The study aimed to include all patients who underwent bariatric surgery in Saudi Arabia while excluding those who had not undergone bariatric surgery and who had preexisting cholelithiasis or cholecystectomy before bariatric surgery.

Sample size calculation

The sample size of this study was calculated using the following formula: 𝒏 = 𝑷(𝟏−𝑷)/ 𝒅𝟐, where n is the sample size, z is the statistic for a level of confidence (95%), p is the anticipated population proportion (50%) for the maximum sample size, and d is precision (5%). The estimated sample size was 385; however, we increased the sample amount from the original 385 to 706.

Questionnaire

The newly developed self‐administered Arabic online questionnaire to determine the incidence of cholelithiasis after bariatric surgery in Saudi Arabia and its associated risk factors was used. The questionnaire tool was constructed after an intensive literature review, which had been done by the researchers, and after taking the surgeon consultation into consideration. The questionnaire consisted of four parts to cover the following. The first part contained the informed consent and the questions related to the participants’ sociodemographic information. The second part focused on questions related to bariatric surgery. The questions related to the incidence of cholelithiasis in bariatric surgery were constituted in the third part. Finally, the questions related to the risk factors of cholelithiasis were covered in the fourth part.

The validity and reliability of the questionnaire were checked by three experts using the content validity index (CVI) and Cronbach’s alpha value, respectively. Regarding validity, the CVI was determined to be 91% by rating each question on a scale of four points according to its relevance and appropriateness. Reliability was checked by carrying out a pilot study to determine Cronbach’s alpha value. By analyzing the response of 22 individuals who participated in the pilot study, Cronbach’s alpha value was found to be 0.791. The questionnaire has been published online via social media platforms by a group of data collectors from July 2022 to September 2022 (see Appendices for the questionnaire).

Data entry and statistical analysis

Data entry was performed using Microsoft Excel 2010 (Microsoft Corp., Redmond, WA, USA), and statistical analysis was done using Statistical Package for the Social Sciences (SPSS) version 21 (IBM SPSS Statistics, Armonk, NY, USA). For analysis, continuous variables were defined by mean±standard deviation (SD), whereas categorical variables were described by frequency and percentage. Pearson’s Chi-square test was used to determine the relationship between categorical variables. A significance value (p) of less than 0.05 was considered statistically significant. A logistic regression model was used to assess the predictive factors for the development of cholelithiasis after bariatric surgery.

## Results

The analysis included data from 706 subjects who underwent bariatric surgery. The sociodemographic characteristics showed that 374 (53%) were males, 674 (95.5%) were Saudi citizens, 473 (67%) were married, 480 (68%) were employed, 488 (69.1%) had a diploma or graduate educational level, and 276 (39.1%) were from the southern province (Table 1). Also, the mean age of the participants was 33.8±10.4 years (non-tabulated).

**Table 1:** Sociodemographic characteristics GERD: gastroesophageal reflux disease, COPD: chronic obstructive pulmonary disease

		Number	%
Gender	Female	332	47
	Male	374	53
Nationality	Saudi	674	95.5
	Non-Saudi	32	4.5
Marital status	Single	194	27.5
	Married	473	67
	Divorced	32	4.5
	Widow	7	1
Job status	Employed	480	68
	Retired	13	1.8
	Student	108	15.3
	Unemployed	105	14.9
Educational level	Uneducated	14	2
	Primary	8	1.1
	Intermediate or secondary	149	21.1
	Diploma or graduate	488	69.1
	Postgraduate	47	6.7
Province	Central province	137	19.4
	Eastern province	99	14
	Northern province	39	5.5
	Southern province	276	39.1
	Western province	155	22
Comorbidities	Diabetes	203	28.8
	Hypertension	237	33.6
	Hyperlipidemia	245	34.7
	GERD	271	38.4
	Anemia	281	39.8
	Thyroid disease	252	35.7
	COPD/asthma	234	33.1
	Chronic heart disease	208	29.5
	Chronic liver disease	196	27.8
	Chronic kidney disease	194	27.5
	Cancer	113	16
	Others	9	1.3

**Figure 1:**
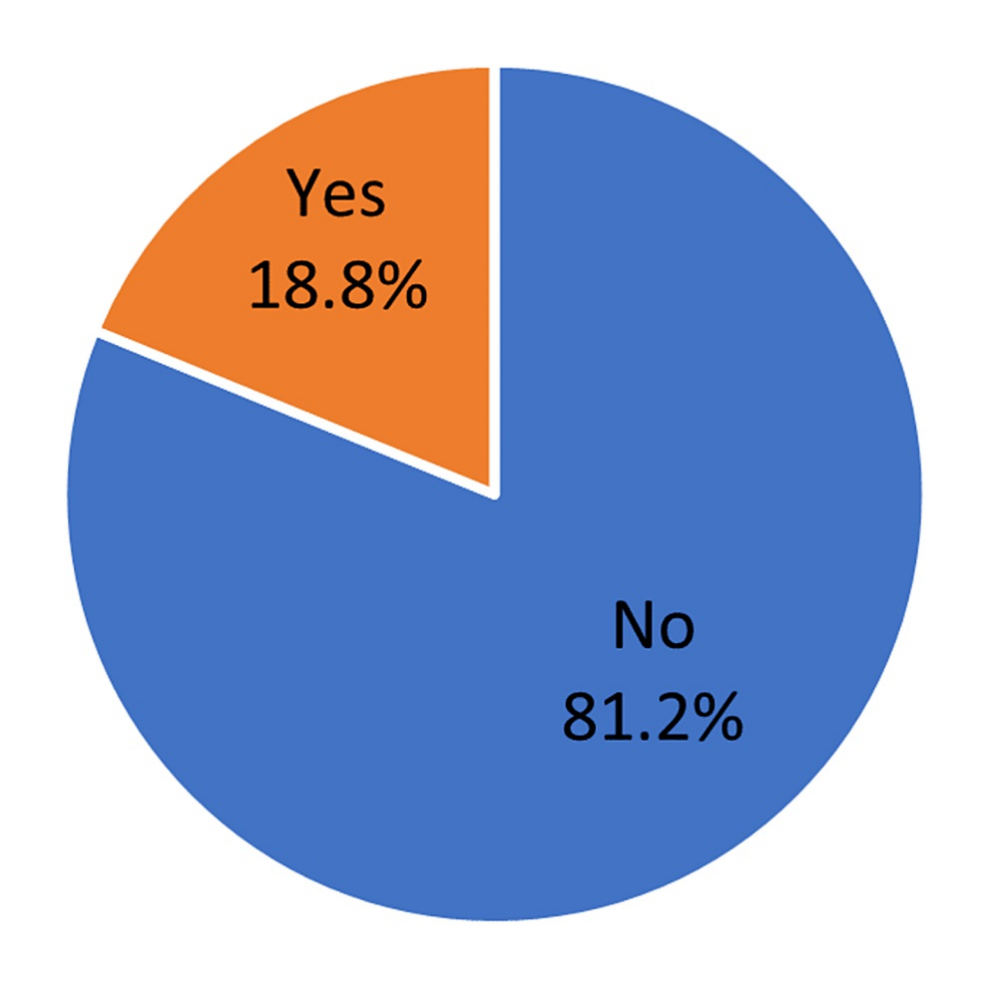
Incidence of gallstones after bariatric surgery

It was found that 133 (18.8%) patients developed cholelithiasis after surgery (Figure 1).

The most commonly undergone bariatric surgery was sleeve gastrectomy (82%), followed by Roux-en-Y gastric bypass (11.3%) and adjustable gastric banding (6.7%). The mean body mass index (BMI) before surgery was 42.3±7.0 kg/m², and one year after surgery, it was 25.4±5.5 kg/m² (Table 2).

**Table 2:** Surgical characteristics and outcomes SD: standard deviation

		Number	%
Type of surgery performed	Sleeve gastrectomy	579	82
	Roux-en-Y gastric bypass	80	11.3
	Adjustable gastric banding	47	6.7
Body mass index (mean±SD)	Before surgery	42.3±7.9	
	After the first three months of surgery	34.7±6.7	
	After six months of surgery	29.6±6.2	
	After one year of surgery	25.4±5.5	

**Figure 2:**
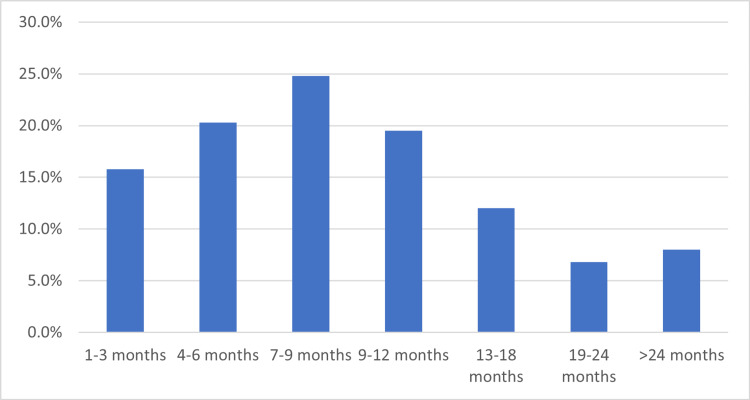
Period of gallstone diagnosis after surgery

It was found that 80.4% of participants develop gallstones within the first year of surgery (Figure 2).

**Figure 3:**
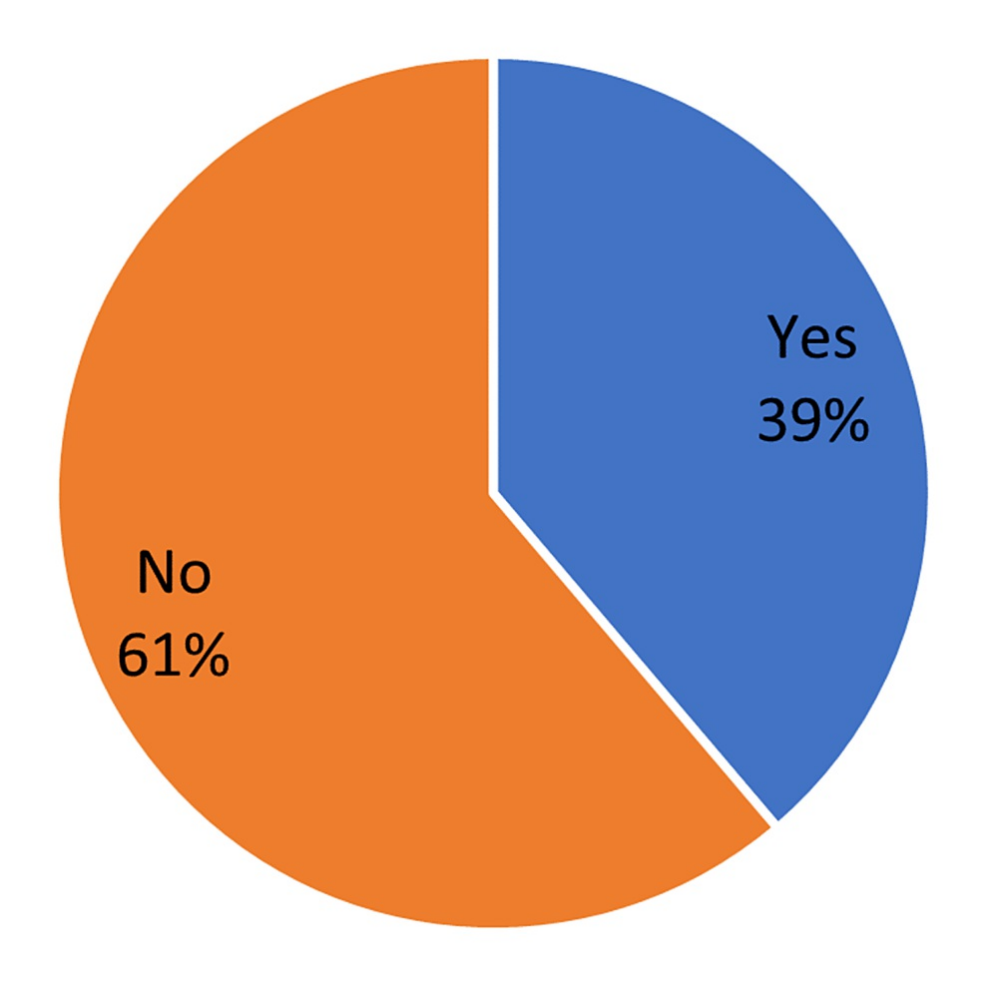
Used medication to prevent the occurrence of gallstones

About 39% of participants use the recommended medication (ursodeoxycholic acid (UDCA) 500-600 mg daily for six months post-surgery) to prevent the occurrence of gallstones (Figure 3).

In comparing the incidence of cholelithiasis between the two genders, the incidence was found to be significantly high in females compared to males (p=0.009). Also, there was no statistically significant difference in the incidence of cholelithiasis between those aged <40 years and ≥40 years (p=0.773). Likewise, the nationality, marital status, and job of the patients did not have a significant association with the incidence of cholelithiasis (p>0.05). However, the incidence was significantly higher among those who had postgraduate educational qualifications (34%) compared to others (p<0.001) (Table 3).

**Table 3:** Relationship between the incidence of cholelithiasis and sociodemographic characteristics

			Incidence of cholelithiasis		P value
			No	Yes	
Gender	Female	Number	256	76	0.009
		%	77.1%	22.9%	
	Male	Number	317	57	
		%	84.8%	15.2%	
Age ≥ 40 years	No	Number	437	103	0.773
		%	80.9%	19.1%	
	Yes	Number	136	30	
		%	81.9%	18.1%	
Nationality	Saudi	Number	545	129	0.348
		%	80.9%	19.1%	
	Non-Saudi	Number	28	4	
		%	87.5%	12.5%	
Marital status	Single	Number	168	26	0.145
		%	86.6%	13.4%	
	Married	Number	374	99	
		%	79.1%	20.9%	
	Divorced	Number	25	7	
		%	78.1%	21.9%	
	Widow	Number	6	1	
		%	85.7%	14.3%	
Job	Employed	Number	384	96	0.116
		%	80%	20%	
	Retired	Number	8	5	
		%	61.5%	38.5%	
	Student	Number	93	15	
		%	86.1%	13.9%	
	Unemployed	Number	88	17	
		%	83.8%	16.2%	
Educational qualification	Uneducated	Number	14	0	<0.001
		%	100%	0%	
	Primary	Number	8	0	
		%	100%	0%	
	Intermediate or secondary	Number	105	44	
		%	70.5%	29.5%	
	Diploma or graduate	Number	415	73	
		%	85%	15%	
	Postgraduate	Number	31	16	
		%	66%	34%	

It was found that the incidence of cholelithiasis was significantly higher in Roux-en-Y gastric bypass (51.2%) and adjustable gastric banding (38.3%) compared to sleeve gastrectomy (12.8%) (p<0.001). It was also observed that patients who had a BMI of >25 kg/m² after one year of surgery significantly had a higher incidence of cholelithiasis (23.1%) compared to those who had a lesser BMI (15.8%) (p=0.014). The incidence of cholelithiasis was significantly lesser in patients who had taken medication to prevent its occurrence (Table 4).

**Table 4:** Incidence of cholelithiasis and its relationship with surgery type and body mass index after one year BMI: body mass index

			Incidence of cholelithiasis		P value
			No	Yes	
Type of surgery	Sleeve gastrectomy	Number	505	74	<0.001
		%	87.2%	12.8%	
	Roux-en-Y gastric bypass	Number	39	41	
		%	48.8%	51.2%	
	Adjustable gastric banding	Number	29	18	
		%	61.7%	38.3%	
BMI one year after surgery	<25 kg/m²	Number	347	65	0.014
		%	84.2%	15.8%	
	≥25 kg/m²	Number	226	68	
		%	76.9%	23.1%	
Used medication to prevent the occurrence of gallstones	No	Number	339	93	0.022
		%	78.5%	21.5%	
	Yes	Number	234	40	
		%	85.4%	14.6%	

A logistic regression model to assess the predictive factors for the development of cholelithiasis after bariatric surgery was performed (Table 5). The model showed that hyperlipidemia (odds ratio (OR)=1.89 (0.94-3.82), p=0.046), cancer (OR=5.00 (1.28-19.52), p=0.021), and Roux-en-Y gastric bypass (OR=3.81 (1.78-8.16), p=0.001) were independently associated with the incidence of cholelithiasis after bariatric surgery. At the same time, the medication to prevent the occurrence of cholelithiasis was found to be a protective factor (OR=0.54 (0.27-0.79), p=0.037).

**Table 5:** Multinomial regression for predicting the risk factors for the incidence of cholelithiasis after bariatric surgery df: degree of freedom, BMI: body mass index, GERD: gastroesophageal reflux disease, COPD: chronic obstructive pulmonary disease

	Wald	df	P value	Odds ratio	95% confidence interval	
					Lower bound	Upper bound
Gender (female)	0.366	1	0.545	0.87	0.56	1.36
Age ≥ 40 years	2.274	1	0.132	1.49	0.89	2.50
BMI ≥ 25 kg/m² one year after bariatric surgery	1.671	1	0.196	0.75	0.49	1.16
Diabetes	1.908	1	0.167	1.58	0.83	3.04
Hypertension	1.489	1	0.222	0.67	0.35	1.28
Hyperlipidemia	3.156	1	0.046	1.89	0.94	3.82
GERD	0.475	1	0.490	1.22	0.70	2.11
Anemia	4.152	1	0.082	0.57	0.34	0.98
Thyroid disease	0.865	1	0.352	0.76	0.42	1.36
COPD/asthma	0.040	1	0.842	1.07	0.55	2.08
Chronic heart disease	0.257	1	0.612	0.82	0.38	1.77
Chronic liver disease	2.523	1	0.112	2.07	0.84	5.10
Chronic kidney disease	0.134	1	0.714	0.85	0.35	2.07
Cancer	5.350	1	0.021	5.00	1.28	19.52
Smoker	0.000	1	0.992	1.00	0.59	1.72
Type of surgery (Roux-en-Y gastric bypass)	11.810	1	0.001	3.81	1.78	8.16
Used medication to prevent gallstone	4.231	1	0.037	0.54	0.27	0.79

## Discussion

Patients who have not been able to sustain weight loss by nonsurgical techniques are candidates for BS because it is based on the assumption that extreme obesity is a condition with various adverse effects on health that may be reversed or improved through effective weight loss. Although BS has been shown to aid in weight loss, there is evidence that it may also have a role in the development of gallstones (GS) [12,22].

The incidence of gallstones or cholelithiasis after BS in our study population was found to be 18.8%. A study done in the Abha region of Saudi Arabia reported an overall incidence of GS after BS to be 61.4%, which is very high compared to our findings [23]. Many other studies have also shown that individuals undergoing BS had a greater incidence of GS development and biliary sludge than the general population, with incidence rates ranging from 10% to 28% [24,25].

In a previous study, gallstone formation rates after BS have been estimated to range from 30% to 53% in a year’s time [26-29]. In our study analysis, 80.4% of the patients who had GS developed it within the first 12 months after BS. This is consistent with the previous study findings, which have shown that the risk of getting GS is highest in the first 12-14 months following BS and remains elevated for the succeeding two years [17,30].

In our study, the incidence of GS was comparatively higher in patients who had a BMI of >25 kg/m² after surgery compared to those who reach a normal BMI. Grover and Kothari [31] reported that the incidence of postoperative cholelithiasis was significantly higher in patients who had a BMI ≥ 40 kg/m².

Some researchers have hypothesized that the type of surgery performed can directly impact the patient’s risk of getting GS. Although sleeve gastrectomy (SG) does not disrupt the biliary contraction mechanisms or the enterohepatic circulation, hypothetically, it should be associated with a reduced incidence of GS formation. However, the supporting evidence for this hypothesis is debatable [10,32]. According to Li et al. [33], symptomatic GS development was observed to be similar between SG and Roux-en-Y gastric bypass (RYGB). Our study findings showed that the incidence was significantly higher in RYGB compared to SG. This is consistent with the findings of Sneineh et al. [34], who also reported an increased incidence of GS in RYGB when compared to SG (14.5% versus 4.4%).

In the general population, variables such as age, obesity, female gender, and parity are known to increase the likelihood of developing GS. This is most likely related to the female sex hormones [35,36]. Our findings showed that the incidence of GS was comparatively higher in females. However, gender and age were not independently associated with the incidence of GS. Our findings are in accordance with other studies, which also reported that gender was not significantly associated with the incidence of GS after BS [24,31].​​​​​​​​​​​​​

Cholesterol gallstone disease is more common in those with metabolic disorders such as hyperlipidemia, type 2 diabetes (T2DM), and insulin resistance [37].​​​​​​​​​​​​​ In our study, we found that the prevalence of hyperlipidemia was found to be independently associated with an increased incidence of GS development. However, we did not find any evidence of an association between GS formation after BS and T2DM and hypertension.

There is no universally accepted method to prevent postoperative GS formation. To date, only two main prophylactic methods have been studied. These include perioperative cholecystectomy and the use of ursodeoxycholic acid (UDCA) with a recommended dose of 500-600 mg daily for six months. We found that the incidence was comparatively lower in individuals who took medication to prevent GS development within six months after surgery. Although there are many positive health outcomes associated with BS, patients should be informed of the potential risk of GS development, and biliary prophylaxis should be considered before surgery [38].​​​​​​​​​​​​​

Our study is the largest to date in Saudi Arabia investigating GS incidence following BS. However, the limitation of this study was the usage of a self-reported questionnaire to collect data, which was prone to recall bias.

## Conclusions

The incidence of gallstones after bariatric surgery was found to be 18.8%. The majority (80.4%) of the affected individuals developed gallstones within 12 months following surgery. Hyperlipidemia and Roux-en-Y gastric bypass surgery were found to be predictive factors for the increased incidence of postoperative gallstone formation; at the same time, the medication used to prevent the occurrence of cholelithiasis was found to be a protective factor. It is necessary to do further prospective studies to determine and gain a better understanding of the influence that the various bariatric procedures have on the modification of stomach emptying and gallbladder motility.

## Appendices

Questionnaire on the incidence of gallstones after bariatric surgery in the Kingdom of Saudi Arabia and its associated risk factors

1. Do you agree to answer this questionnaire, knowing that all information will remain confidential and will only be used by researchers in this research?

- Yes

- No

2. Have you had weight loss surgery before?

- Yes

- No (questionnaire ends)

Personal Data

(Please fill in the required data accurately.)

1. Gender:

- Male

- Female

2. Age:

- Open question

3. Nationality:

- Saudi

- Non-Saudi

4. Place of residence:

- Central region

- Eastern region

- Western region

- Southern region

- Northern region

5. Educational level:

- Uneducated

- Elementary

- Intermediate or secondary

- College

- Postgraduate

6. Marital status:

- Single

- Married

- Divorced

- Widowed

7. Job:

- Student

- Employee

- Not employed

- Retired

Questions Related to Obesity Surgery

(Please fill in the required data accurately.)

1. When did you perform bariatric surgery? (Please choose the easiest date form for you.)

- Month/year (Gregorian date)

- Month/year (Hijri date)

2. What kind of operation did you have?

- Sleeve gastrectomy

- Roux-en-Y gastric bypass (gastric bypass surgery)

- Adjustable gastric banding (gastric banding surgery)

- Other (Please specify.)

3. Weight before surgery (in kilograms):

4. Weight after the first three months of surgery (in kilograms):

5. Weight after six months of surgery (in kilograms):

6. Weight after one year of surgery (in kilograms):

7. Length (in centimeters):

Questions Related to the Incidence of Gallstones After Bariatric Surgery

(Please fill in the required data accurately.)

1. Were you diagnosed with gallstones or had an operation to remove your gallbladder before your bariatric surgery?

- Yes

- No

2. Were you diagnosed with gallstones after undergoing bariatric surgery?

- Yes

- No

3. If the answer to the previous question is yes, when did you get gallstones after the surgery?

- 1-3 months

- 4-6 months

- 7-9 months

- 9-12 months

- 13-18 months (a year and a half)

- 19-24 months (two years)

Risk Factors Questions

1. Do you have any of the following diseases? (You can choose more than one answer.)

- Diabetes

- Hypertension

- Hyperlipidemia

- Gastroesophageal reflux disease (GERD)

- Anemia

- Thyroid diseases

- Asthma or chronic lung disease

- Chronic heart disease

- Chronic liver disease

- Chronic kidney disease

- Cancer

- Other (Please specify.)

- None

2. Are you a smoker?

- Yes

- No

3. Did you use any medications to prevent or break up gallstones during the first six months after the operation?

- Yes

- No

Thank you for your time and cooperation. We wish you a good, healthy life.

## References

[1] Blüher M (2019). Obesity: global epidemiology and pathogenesis. Nat Rev Endocrinol.

[2] (2023). World Health Organization: Prevalence of obesity among adults, BMI >= 30 (age-standardized estimate) (%). https://www.who.int/data/gho/data/indicators/indicator-details/GHO/prevalence-of-obesity-among-adults-bmi-=-30-(age-standardized-estimate)-(-).

[3] Altaf A, Abbas MM (2019). Public perception of bariatric surgery. Saudi Med J.

[4] Chang SH, Stoll CR, Song J, Varela JE, Eagon CJ, Colditz GA (2014). The effectiveness and risks of bariatric surgery: an updated systematic review and meta-analysis, 2003-2012. JAMA Surg.

[5] Alfadhel SF, Almutairi HS, Al Darwish TH, Almanea LT, Aldosary RA, Shook AH (2020). Knowledge, attitude, and practice of bariatric surgery among adult Saudi community, Saudi Arabia, 2019. J Family Med Prim Care.

[6] Albaugh VL, Banan B, Ajouz H, Abumrad NN, Flynn CR (2017). Bile acids and bariatric surgery. Mol Aspects Med.

[7] Miras AD, le Roux CW (2013). Mechanisms underlying weight loss after bariatric surgery. Nat Rev Gastroenterol Hepatol.

[8] Welbourn R, Hollyman M, Kinsman R (2019). Bariatric surgery worldwide: baseline demographic description and one-year outcomes from the fourth IFSO global registry report 2018. Obes Surg.

[9] Desbeaux A, Hec F, Andrieux S, Fayard A, Bresson R, Pruvot MH, Mulliez E (2010). Risk of biliary complications in bariatric surgery. J Visc Surg.

[10] Manatsathit W, Leelasinjaroen P, Al-Hamid H, Szpunar S, Hawasli A (2016). The incidence of cholelithiasis after sleeve gastrectomy and its association with weight loss: a two-centre retrospective cohort study. Int J Surg.

[11] Boerlage TC, Haal S, Maurits de Brauw L (2017). Ursodeoxycholic acid for the prevention of symptomatic gallstone disease after bariatric surgery: study protocol for a randomized controlled trial (UPGRADE trial). BMC Gastroenterol.

[12] Alsaif FA, Alabdullatif FS, Aldegaither MK, Alnaeem KA, Alzamil AF, Alabdulkarim NH, Aldohayan AD (2020). Incidence of symptomatic cholelithiasis after laparoscopic sleeve gastrectomy and its association with rapid weight loss. Saudi J Gastroenterol.

[13] Alimoğulları M, Buluş H (2020). Predictive factors of gallstone formation after sleeve gastrectomy: a multivariate analysis of risk factors. Surg Today.

[14] Plecka Östlund M, Wenger U, Mattsson F, Ebrahim F, Botha A, Lagergren J (2012). Population-based study of the need for cholecystectomy after obesity surgery. Br J Surg.

[15] Jonas E, Marsk R, Rasmussen F, Freedman J (2010). Incidence of postoperative gallstone disease after antiobesity surgery: population-based study from Sweden. Surg Obes Relat Dis.

[16] Andrés-Imaz A, Martí-Gelonch L, Eizaguirre-Letamendia E, Asensio-Gallego JI, Enríquez-Navascués JM (2021). Incidence and risk factors for de novo cholelithiasis after bariatric surgery. Cir Esp (Engl Ed).

[17] Talha A, Abdelbaki T, Farouk A, Hasouna E, Azzam E, Shehata G (2020). Cholelithiasis after bariatric surgery, incidence, and prophylaxis: randomized controlled trial. Surg Endosc.

[18] Wan Q, Zhao R, Chen Y, Wang Y, Wu Y, Wu X (2021). Comparison of the incidence of cholelithiasis after sleeve gastrectomy and Roux-en-Y gastric bypass: a meta-analysis. Surg Obes Relat Dis.

[19] Aldriweesh MA, Aljahdali GL, Shafaay EA (2020). The incidence and risk factors of cholelithiasis development after bariatric surgery in Saudi Arabia: a two-center retrospective cohort study. Front Surg.

[20] El Shafey HE, Elgohary H, El Azawy M, Omar W (2021). The incidence of gall stones after bariatric surgery and its association with weight loss. Int J Surg Open.

[21] Makki AM, Aldaqal SM (2016). Prevalence and management of gall stones in sleeve gastrectomy. Br J Med Res.

[22] Wolfe BM, Kvach E, Eckel RH (2016). Treatment of obesity: weight loss and bariatric surgery. Circ Res.

[23] Shubayr N, Elbashir M, Alashban Y (2022). Incidence of gallbladder stone formation after bariatric surgery using ultrasound imaging in the southern region of Saudi Arabia. Cureus.

[24] Hasan MY, Lomanto D, Loh LL, So JB, Shabbir A (2017). Gallstone disease after laparoscopic sleeve gastrectomy in an Asian population-what proportion of gallstones actually becomes symptomatic?. Obes Surg.

[25] Festi D, Dormi A, Capodicasa S (2008). Incidence of gallstone disease in Italy: results from a multicenter, population-based Italian study (the MICOL project). World J Gastroenterol.

[26] Shiffman ML, Shamburek RD, Schwartz CC, Sugerman HJ, Kellum JM, Moore EW (1993). Gallbladder mucin, arachidonic acid, and bile lipids in patients who develop gallstones during weight reduction. Gastroenterology.

[27] Shiffman ML, Sugerman HJ, Kellum JH, Brewer WH, Moore EW (1993). Gallstones in patients with morbid obesity. Relationship to body weight, weight loss and gallbladder bile cholesterol solubility. Int J Obes Relat Metab Disord.

[28] Iglézias Brandão de Oliveira C, Adami Chaim E, da Silva BB (2003). Impact of rapid weight reduction on risk of cholelithiasis after bariatric surgery. Obes Surg.

[29] Dhabuwala A, Cannan RJ, Stubbs RS (2000). Improvement in co-morbidities following weight loss from gastric bypass surgery. Obes Surg.

[30] Vural A, Goksu K, Kahraman AN, Boy FN, Anil BS, Fersahoglu MM (2020). Increased gallstone formation after sleeve gastrectomy and the preventive role of ursodeoxycholic acid. Acta Gastroenterol Belg.

[31] Grover BT, Kothari SN (2014). Biliary issues in the bariatric population. Surg Clin North Am.

[32] Melissas J, Koukouraki S, Askoxylakis J, Stathaki M, Daskalakis M, Perisinakis K, Karkavitsas N (2007). Sleeve gastrectomy: a restrictive procedure?. Obes Surg.

[33] Li VK, Pulido N, Martinez-Suartez P, Fajnwaks P, Jin HY, Szomstein S, Rosenthal RJ (2009). Symptomatic gallstones after sleeve gastrectomy. Surg Endosc.

[34] Sneineh MA, Harel L, Elnasasra A (2020). Increased incidence of symptomatic cholelithiasis after bariatric Roux-en-Y gastric bypass and previous bariatric surgery: a single center experience. Obes Surg.

[35] Li VK, Pulido N, Fajnwaks P, Szomstein S, Rosenthal R, Martinez-Duartez P (2009). Predictors of gallstone formation after bariatric surgery: a multivariate analysis of risk factors comparing gastric bypass, gastric banding, and sleeve gastrectomy. Surg Endosc.

[36] Cirillo DJ, Wallace RB, Rodabough RJ, Greenland P, LaCroix AZ, Limacher MC, Larson JC (2005). Effect of estrogen therapy on gallbladder disease. JAMA.

[37] Portincasa P, Moschetta A, Palasciano G (2006). Cholesterol gallstone disease. Lancet.

[38] Altieri MS, Yang J, Nie L, Docimo S, Talamini M, Pryor AD (2018). Incidence of cholecystectomy after bariatric surgery. Surg Obes Relat Dis.

